# Georgia State Dental Society

**Published:** 1889-12

**Authors:** 


AMERICAN JOURNAL

—OF—

DENTAL SCIENCE.

Vol. xxiii. Baltimore, December, 1889. No. 8.

ARTICLE I.

### GEORGIA STATE DENTAL SOCIETY.

### TWENTY-FIRST ANNUAL MEETING.

[Reported by "Mrs. M. W. J."]

The Georgia State Dental Society held its twenty-first annual meeting at Tybee Island, near Savannah, June eleventh to fifteenth, 1889.

The meeting was called to order, for organization at 12 o'clock M., Tuesday, June 11, the President, Dr. S. A. White, in the chair. In the absence of the Recording Secretary, (Dr. H. H. Johnson), Dr. D. D. Atkinson was appointed Secretary *pro tem.* The Corresponding Secretary, Dr. L. D. Carpenter, and the Treasurer, Dr. H. A. Lowrance, reported ready for duty. The Executive Committee asked for further time. A short recess was taken for payment of dues.

The Society was then called to order again, the annual address of the President being the first order of business.

The President made a brief verbal address, stating that owing to protracted ill-health it had been impossible for him to prepare a formal address.

On motion of Dr. H. S. Colding, the explanation was accepted and the President excused.

The Executive Committee reported the following names as recommended for active membership: J. H. Boozer, Cuthbert, Ga.; L. W. Daniels, Savannah, Ga.; T. S. Daniels, Wadley, Ga.

On motion, the Secretary was instructed to cast the ballot, and they were declared duly elected.

Dr. Carpenter read a letter from Vice-President J. A. Thornton, regretting his inability to be present, owing to sickness in his family.

Dr. W. G. Browne was placed upon the Executive Committee, replacing an absent member.

A report from the Executive Committee, recommending the hours from 9 A. M. to 1, and from 3 to 5:30 P. M., for meetings, with clinics before, between, and after these hours, was discussed at some length. After various suggestions from Drs. Browne, Catching, Colding, Adair, Holland, and others, the resolution was amended, and the hours of from 8.30 to 12:30 for daily scientific meetings with clinics at 3 P. M. Adopted.

Dr. B. H. Catching made a brief address on the subject of the value of membership in the Georgia State Dental Society, a society which stands high in the profession, the reports of its proceedings being quoted in all the leading journals as authoritative in matters pertaining to dentistry.

The newly elected members were invited to express their views upon the subject.

Dr. Boozer responded that though taken rather by surprise in this suggestion, he fully appreciated the honor which had been conferred upon him, and expected to reap the advantages implied. Any member of the profession practicing in the State of Georgia, and not joining the State Society, was not truly in line. Membership would inevitably create a greater desire for progress, through the spirit of emulation.

Dr. J. W. Daniel said that he had been a member of the Society in former years, and had only lost his membership through prolonged absence from the United States. He was glad to rejoin the Society, and hoped to be able to attend regularly meetings which cannot be otherwise than pleasant and beneficial.

Dr. T. S. Daniels also reported briefly.

Dr. R. B. Adair commented upon the absence of newspaper reporters, and said that he considered it a matter of importance in the education of the people. On the train he had met a man of ordinary intelligence in scientific matters, but whose only idea of the annual meeting was that it was for the purpose of *regulating prices!* but who seemed very much interested when the real object of the meeting, and its humanitarian work, was made clear to him.

He thought that a synopsis of such portions of the proceedings as were of general interest, should be prepared daily. He therefore moved that "Mrs. M. W. J.," the reporter for the Society, be instructed to prepare such a synopsis for newspaper publication.

Dr. H. S. Colding said he had promised to give such notes to a Savannah daily paper.

The motion was voted upon and carried unanimously.

(The synopsis was duly furnished at the close of each session. REPORTER.)

On motion of Dr. Browne, adjourned.

---

Wednesday June 12. Called to order at 8:30 A. M., the President in the chair. After a short recess for payment of dues and reading of the minutes of Tuesday's session, the following names were recommended for membership by the Executive Committee: F. R. Parramore, Valdosta, Ga.; B. W. Cubbege, Savannah, Ga., and they were duly elected.

Dr. W. G. Browne said that he wished to ask the unanimous consent of the Society that young men, paying their license of $10.00, be considered members of the Society at once. They would then feel identified with the Society, and would probably always remain members. But if they were allowed to drift away, feeling that no interest was taken in them, they would very likely never be heard of again in the Society. Let them come right in, as members, and make them realize the good to be gained.

Dr. Catching thought this privilege should be extended only to those who passed the Examining Board successfully.

Dr. L. D. Carpenter thought they could only come in on the recommendation of the Executive Committee.

The President stated that such a motion being virtually an amendment to the By-Laws of the Society, must be made in writing, and hoped that action would be taken promptly, that it might be included in the new edition now being printed.

Dr. Catching called attention to the fact that a single negative would kill an amendment to the By-Laws, and moved that the amendment lie over for one year.

Dr. Gale thought it such an honor to belong to the Society that it was at least worth the effort of raising the additional $5.00 necessary for the regular initiation fee.

The President said that the object of the amendment was to assist and encourage young men, who have just been under heavy expenses in getting through College, fitting up an office, traveling to the Society to pass an examination, etc. Under such circumstances, to a young man just starting out in life, $5.00 is sometimes a large sum; and more than that, and behind the money question is the moral support given a young man by this act of help and encouragement.

One of the main objects of the Society is to help the younger members of the profession, and this is one way in which it can be done. They have paid their college dues, and their board bills, and their license and traveling expenses, and hotel bill here, and we can easily afford to remit this other $5.00. Let us take them by the hand and say: "Come in and be one of us." Give them that much encouragement, and show our appreciation of their good intention in coming here.

Dr. Boozer thought that enrolling them in this way would add so many to the list of members that their future annual dues would more than compensate for the loss of their initiation fees.

Dr. Catching said it should not be made a question of dollars and cents. It is a question of building up the Dental profession in the State of Georgia. Bringing them in in this way, they will at once conform to the spirit of the Constitution and By-laws of the Society, and grow up with the interests of the profession at heart.

Dr. Wardlaw said, that from his own knowledge he could state that unless this becomes a law the Society would lose one of the brightest and most useful members the profession will ever have. He is prepared to go in now with his whole soul, and will soon put our older members to the blush. Otherwise he will go home disheartened, and probably never meet with us again.

Dr. Carpenter endorsed the resolution most heartily.

Dr. Thompson said it was virtually abolishing the initation fee; but if the Society could afford it, he was willing. Many would never come back, and he would get no annual dues from them, besides losing their initiation fee, and from what he had heard he believed the treasury was low, as it cannot afford to publish a volume of Transactions—a most desirable thing. He would suggest that we give them time, and let them pay later, when better able.

Dr. Whitaker said that of the very large number licensed to practice since the passage of the law, but a very small percentage have joined the Society.

Dr. D. D. Atkinson said that the new members so far elected are all men who have been practicing for a number of years—from four up. They are not "young men." At Cumberland Island the same question was brought up, and the young men to be benefited by it declined to receive the gift, and they are now an honor to the profession. If they pay $10.00 for a privilege, they appreciate it more than when it comes as a gift. But the resolution itself would do honor to the Society, and he was in favor of it.

Dr. Gayle said he would vote for the resolution if made operative for one year only.

Dr. Catching said it would be made clear that the rule applies only to young students, not to those old mossbacks who come up and pass the Board only because they are forced to do so by the law.

Dr. Sid. Holland thought abolishing fees established a bad precedent. If a young man has not got natural ambition, you cannot put it into him by *fostering* him. He said he was always ready to lend a helping hand, to tell a young man all he knew, but that if he had ambition, and was determined to succeed, he would succeed anyhow.

The Dental Society is not for the young men alone. Like a bunch of keys in the pocket, which does not rust, where one alone would be eaten up; friction brings out sparks. If anything is to be abolished, it should be the Examining Board. There are two laws on the subject, which conflict; one says you shall do a thing, the other says you shall not. One law says the Dental Department of the Southern College shall graduate a man and give him the right to practice dentistry in the State of Georgia; another law says he has no right to practice dentistry in the State of Georgia until he has passed this Examining Board. The State ought to be ashamed of such laws.

Dr. R. B. Adair—The Society is almost unanimous on this question, but one man can vote it down.

Dr. Sid. Holland—I say as Aleck Stephens said of secession, " Georgia is wrong, but I am with her."

Dr. Lowrance thought if anything was abolished it should be the fee paid to the Examining Board.

Dr. R. B. Adair said that no matter how ambitious a young man might be to become a member of the Society, he must have the money, too. One young man borrowed the money to come here on, because it was a thing that he had to do, but he would not pay this extra five dollars. This resolution does not take anything out of the treasury, as we have all we would have if they did not join, while next year we have the probability of collecting their annual dues.

On motion of Dr. Catching, the resolution was laid on the table.

### REPORTS OF STANDING COMMITTEES.

### PHYSIOLOGY AND ETIOLOGY.

Dr. W. C. Wardlaw, chairman of the committee, read a paper entitled :

### ETIOLOGY OF CARIES.

At the conclusion of the reading, Dr. B. H. Catching, opening the dicussion, said: That paper alone pays me for coming to Tybee. It is a common sense, plain setting forth of the causes of decay. At first I set my face against the germ theory. I was one of those who called them " bugs." But it has been forced upon us ; it is the only feasible and reasonable explanation of all the features of the case, and it has been made so clear that all can understand and appreciate it.

Dr. R. B. Adair—There is too much in the paper for all of it to be discussed at one session. I was like Dr. Catching in my opposition to the germ theory. We cannot all be microscopists ; we have to accept the evidence placed before us by others. Scientific men like Black, Hietzman, Bodecker, and, above all, Dr. Miller, of Berlin, give us their positive testimony after having demonstrated these things to themselves, throwing a clear, bright light on what was once the most obscure subject we had to deal with. One practical point is that we should sterilize every cavity and destroy the micro-organisms that cause decay. The most effectual agents for that purpose are bichloride of mercury, carbolic acid, or creosote, and oil of cloves.

Dr. W. G. Browne—If all decay is thoroughly removed, will not that be sufficient?

Dr. Adair—Probably not. Other organisms will be introduced. But this is not the time for the discussion of operative dentistry. It was at first difficult for me to see how these minute organisms could bore through the dense enamel, but Dr. Miller has shown us that it is the secretions, or the excreta, effete matter, the bacteria, that dissolve the tooth substance, making an entrance for other bacteria.

Dr. Brown—I have accepted this theory from the moment it was made partially clear to me; but I do not yet see why it is that in persons of certain temperament and constitution the teeth decay so much more rapidly than with others.

Dr. Holland—Dr. Wardlaw's paper is, as the old Roman lawyer said, *utile dulce*—both useful and good, sweet to the ear—and it is truth from beginning to end. The various theories heretofore advanced are all summed up and included in the germ theory, which is in reality a chemical theory and a vital theory. Where do the microbes come from? They exist everywhere; in the air, the earth, the water. Miller wondered how many there were in a cubic foot of air in the polluted air of Paris, and in the pure air on a high mountain-top. In the mouth they are propagated by warmth and moisture.

President S. A. White (Dr. Carpenter in the chair in the absence of both Vice-Presidents)—What starts decay in the tooth? What does it begin upon when the tooth is in its condition of integrity?

Dr. Holland—Did Diety intend that his creatures should be a failure? If he was perfect he would have remained perfect.

Dr. Carpenter—These germs of imperfection came in when sin came in.

Dr. Adair—I move we rule out all theological questions.

Dr. Holland—Man was made perfect, and if he had remained perfect he would never have wanted a dentist. We ourselves produce these germs, but beyond that we know nothing. We cannot now find a perfect tooth with no taint from the past. The tooth is but calcium carbonate; apply hydrochloric acid, and we have its destruction. Shall we go back and live in tents and herd cattle that we may have more perfect teeth? No! But let us banish condiments, prepare our food more simply, live more in the open air, take more precautions as to cleanliness. It has been said that absolute cleanliness will absolutely prevent decay, but I don't believe that, because we find decay on the most prominent points where the motion of the lips keeps the tooth clean. We don't know all about it yet!

Dr. McElhaney wished to endorse Dr. Wardlaw's paper; said he had given much study to the subject, which is now deemed of such importance that the Illinois State Society has engaged the entire time of Dr. Black to be devoted exclusively to the investigation of this subject. Such men as Miller are at work on the right line; it is the line for us to work on if we would prevent decay.

Dr. White—I do not believe in the idea of infection from one tooth to another. I believe that each cavity is dependent upon itself and its own germ except in approximate surfaces. Why, when we find decay on one side of the mouth, do we look for and expect to find decay in the corresponding teeth on the other side? Simply because they were developed at the same time and under the same influences. Dr. White considers bichloride of mercury a dangerous agent for use in the mouth; carbolic acid is equally reliable and can always be used with safety. For fifteen years he has prescribed as a mouth-wash—

| | | |
|---|---|---|
| Carbolic acid crystals, | - - | 4 drachms. |
| Glycerine, | - - - - | 3 oz. |
| Water, | - - - - | 3 oz. |

A few drops to be used on a soft tooth-brush. He is satisfied from the record kept that those who use this mouth-wash habitually will have much less work done on the teeth than others.

Dr. Catching—Wherever we find decay, examination with the microscope will show a pre-existing lodgement for germs. The corresponding decay on opposite sides of the mouth spoken of, is due to corresponding conditions of nutrition and assimilation—the teeth are constitutionally alike. We will often find an approximal decay in a first class molar when the bicuspid in contact is not decayed, because the two teeth were not developed at the same time; they are not constitutionally alike.

Dr. Carpenter—We know but little of the real causes of decay. Investigations have opened the door for us, but we must work for ourselves—we must go back further—

Dr. Catching—Dr. Holland went back to Adam and Eve.

Dr. Carpenter—But for Adam's eating the forbidden fruit we should have neither death nor decay; but as it is, we have predispositions which we are always fighting to overcome—that's where it all comes from. Chemistry and bugs alike go back to the same starting point.

Dr. McElhaney—The acid in the apple that Adam ate?

Dr. Browne—No; the bugs and worms in the apple.

Dr. Wardlaw, in closing the discussion, said that several points had been raised and questions asked to which he wished to reply. He was very glad the scales had been removed from his eyes. At first he thought the germ theory far-fetched, and paid but little attention to it, until, at Louisville, Dr. Sudduth's remarks as to what has been actually shown under the microscope awoke his attention and caused him to study it more carefully. He now considers that it meets all requirements. He was astonished to hear Dr. Carpenter say we know so very little about it, when we have had such clear mathematical and chemical demonstration. Every other cause being eliminated, we find all the essentials here. Dr. Catching asked about abrasion—is it due to chemical action? Is it caused by micro-organisms? Lactic acid don't do everything; organic acids will destroy organic elements. Micrococci make the cavity, penetrating from the point of lodgment. Mineral acids help in abrasion. A well formed tooth, isolated, will not decay unless through the action of mineral acids or organic defect, lack of thorough calcification. I have seen a case of decay on the point of a cuspid, where microscopic examination would probably have revealed a minute fracture, or other defect in organization. Lactic acid requires twenty-four hours for its perfect work upon the teeth; the point of lodgment must therefore be protected. As to *infection*, the germs float around in the fluids of the mouth, ready to lodge where a good place is found, as in fermenting food. Dr. Holland says oxygen will inevitably decompose a dead body. I say if sterilized—all germs excluded—it will not decompose. Germs consume oxygen while at work, but unless you have the germs you will not have decomposition.

Dr. Brown asked about the different degrees of decay in certain months, with different temperaments, different constitutions, etc. I think it is due to incidental circumstances. Some teeth afford no lodgment for offensive matter; others are so shaped as to favor its retention. It may also depend upon the condition and character of the fluids of the mouth, which in some conditions promote fermentation.

Dr. Holland—We have cases of spontaneous abrasion, where the surface is as smooth as a piece of glass. In decay through chemical action the surface is rough. We must have different agents to produce such different results. I take the ground that there can be no degeneration or decomposition without the presence of oxygen, and do not believe either Miller or Black can prove the contrary.

On motion, the subject was passed.

A letter was read by the Secretary from Dr. E. M. Allen, expressing his appreciation of the resolutions in his favor passed at Dalton. Dr. Allen is one of the three survivors of those who organized the Society in 1869.

A letter was read from Dr. Ottofy, of Chicago, chairman of a committee appointed by the Illinois State Dental Society, to raise a fund to defray the expense of the examination and tabulation of the condition of prehistoric skulls in the various museums in this country. The letter stated that the Illinois Society would be responsible for the judicious expenditure of this fund, to which it had appropriated \$100.00, and asks for contributions from other State Societies. This committee consists of Drs. Louis Ottofy, J. M. Crome, and G. H. Cushing.

On motion of Dr. Catching, the Secretary was instructed to make a suitable reply to this communication, expressing the appreciation of the Georgia State Society of the efforts being made in this line, and authorizing the committee to draw on the Treasurer for the sum of \$25.00.

A circular letter from Dr. J. N. Crouse, relating to the work of the Dental Protective Association, was read.

On motion of Dr. Coyle, the endorsement of the Georgia State Society was given to the work of the Dental Protective Association, and the members of the Society recommended to give their individual support by sending their \$10.00 subscriptions.

A letter from the venerable Dr. E. Parsons, now prostrated on what will probably prove to be his death bed, was read, and, on motion of Dr. Catching, ordered spread upon the minutes. On motion of Dr. Thompson, Drs. Carpenter, Holland, and Lowrance were appointed a committee to wait upon Dr. Parsons and express to him the sympathy of the Society in his illness.

Drs. Hopps, Catching, Browne, White and others spoke of work done by Dr. Parsons, now in his 83d year, in building up the profession and elevating its standard. Dr. Browne spoke of fillings inserted by Dr. Parsons over forty years ago, which are still in good condition.

Reports of standing committees resumed.

PATHOLOGY AND THERAPEUTICS.

Dr. J. H. Coyle read a paper on this subject.

Dr. Catching said that Dr. Coyle's paper covers so nearly the ground previously occupied by Dr. Wardlaw that a discussion could only bring out the same points.

Dr. R. B. Adair spoke of the hyperæmic condition of the gums described by Dr. Coyle as a cause of decay, and said that, according to his observation, that very condition produced a cessation of decay, and that he could not assent to Professor Coyle's position.

Dr. Wardlaw thought that the last speaker assumed a condition of the fluids the very opposite to that described by the essayist. In pyorrhœa alveolaris, and when the gums are congested, it will be found that the secretions are alkaline, the condition described by Dr Coyle as conducing to decay being a secretion of sticky, slimy mucus, easily recognized. He thought that Dr. Adair and Dr. Coyle had in mind exactly opposite conditions. He agreed entirely with Prof. Coyle, and was glad to find that so many were studying pathological conditions rather than merely filling teeth. He thought Dr. Coyle presented methods of treatment exactly the right thing. We will all go further back in our treatment as we study this question more. In pregnant women he prescribes the free use of limewater as a mouthwash. He thought that Prof. Coyle did not mean to be understood as saying that acids lodged in the dentine as a starting point.

Prof. Coyle replied, that his paper, being very brief, he had dealt only in general terms. It was well understood that microbes cannot penetrate the enamel; they must find a preceding lodging place. Hereditary conditions, malnutrition, or some other imperfection of the enamel, enables them to reach the dentine, which thus becomes the starting point for their active operations. They never break through sound enamel. Through congenital defects, interrupted nutrition, etc., acids penetrate, perhaps, through the merest microscopic fissure, decomposing the dentine until the enamel gives way, and the whole tooth seems to be crushed by the merest accident. Dr. Adair spoke of quite another condition. I did not allude to the specific condition pyorrhœa alveolaris—a subject which we will not go into at present. As practitioners we do not sufficiently recognize the sequences of acid conditions. We look for cavities and fill them, perhaps throwing out some mere suggestion as to the condition of the gums; but we do not give sufficient attention to these conditions before operating.

On motion of Dr. Colding, the subject was passed.

Dr. L. Carpenter requested all those present to give in the names of all practicing dentists within their knowledge for publication in the new directory.

On motion, adjourned.—*Southern Dental Journal.*

[*To be continued.*]

